# Clinical Significance of Ventricular Tachyarrhythmias in Patients Undergoing Valve Replacement: A Nationwide Population-Based Study

**DOI:** 10.3389/fcvm.2021.676897

**Published:** 2021-07-15

**Authors:** Guan-Yi Li, Yun-Yu Chen, Kuo-Liong Chien, Yenn-Jiang Lin, Tzu-Ting Kuo, Fa-Po Chung

**Affiliations:** ^1^Division of Cardiology, Department of Medicine, Taipei Veterans General Hospital, Taipei, Taiwan; ^2^Institute of Epidemiology and Preventive Medicine College of Public Health, National Taiwan University, Taipei, Taiwan; ^3^Institute of Clinical Medicine and Cardiovascular Research Center, National Yang-Ming University, Taipei, Taiwan; ^4^Division of Cardiovascular Surgery, Department of Surgery, Taipei Veterans General Hospital, Taipei, Taiwan; ^5^Department of Medicine, School of Medicine, National Yang Ming University, Taipei, Taiwan

**Keywords:** bioprosthetic valve, implantable cardioverter-defibrillator, mechanical valve, valve replacement, ventricular tachyarrhythmia

## Abstract

**Background:** The clinical significance and outcomes of ventricular tachyarrhythmias (VTa) in patients undergoing valve replacement have rarely been reported.

**Objective:** This study aimed to investigate the incidence and outcome of VTa after surgical valve replacement.

**Methods:** We conducted a population-based retrospective cohort study using data obtained from the Taiwan National Health Insurance Research Database. In total, 10,212 patients were selected after 1:1 propensity-score matching based on the type of prosthetic valve used (mechanical vs. bioprosthetic). Various outcomes during long-term follow-up were analyzed.

**Results:** After a median follow-up period of 59.6 months, the crude incidence rate of VTa after surgical valve replacement was 4.8/1,000 person-years, and the cumulative incidence of VTa persistently increased after surgery. Furthermore, the occurrences of VTa after valve replacement significantly increased the risk of cardiovascular (CV) death (*P* < 0.001, HR 1.67, 95% CI 1.41–1.96), stroke- (*P* < 0.001, HR 1.66, 95% CI 1.37–2.01), atrial fibrillation- (*P* < 0.001, HR 2.80, 95% CI 2.42–3.24), and congestive heart failure-related hospitalization (*P* < 0.001, HR 2.61, 95% CI 2.30–2.95). Among patients with VTa, all-cause mortality (*P* = 0.001, HR 0.49, 95% CI 0.32–0.75) and CV death (*P* = 0.047, HR 0.58, 95% CI 0.34–0.99) in those with implantable cardioverter-defibrillator (ICD) implantation were lower than those without.

**Conclusion:** The crude incidence rate of VTa after surgical valve replacement was 4.8/1,000 person-years, and the cumulative incidence of VTa persistently increased during follow-up. The presence of VTa after surgical valve replacement increases hospitalization and CV death, while ICD implantation reduced the mortality rate in these patients.

## Introduction

Ventricular tachyarrhythmias (VTa), including ventricular tachycardia (VT) and ventricular fibrillation (VF), often contribute to worse clinical outcomes in patients with structural heart disease, such as valvular heart disease (VHD) (1). Of note, VHD might lead to structural remodeling, which may result in myocardial stretching and scar formation and consequently increase the probability of ventricular arrhythmogenesis (2). Despite surgical valve replacement, patients could still be at risk of sudden cardiac death due to arrhythmias, progressive myocardial dysfunction, and valve-related complications (3). This finding was supported by the fact that arrhythmias account for 6% of deaths in 1,533 patients who underwent aortic or mitral valve replacement (4). However, only a few studies focused on the incidence of VTa after valve replacement and associated clinical outcomes. This study aimed to assess the incidence of VTa after surgical valve replacement, the correlation between prosthetic valves and VTa, and the clinical impact of VTa on various outcomes, including mortality and hospitalization, using a nationwide population-based database. Furthermore, we investigated the role of implantable cardioverter-defibrillator (ICD) implantation in these patients. We believe that the abovementioned findings could improve the understanding of the clinical significance of VTa after valve replacement.

## Materials and Methods

### Study Design and Participants

We conducted a population-based retrospective cohort study, and data were collected from January 1, 2000, to December 31, 2011, using the National Health Insurance Research Database (NHIRD). Patients receiving their first surgical valve replacement were grouped based on the procedural codes of the Specifications of the National Voluntary Consensus Standards for Cardiac Surgery: bioprosthetic valve replacement (procedure codes 35.21, 35.23, 35.25, and 35.27) and mechanical valve replacement (procedure codes 35.22, 35.24, 35.26, and 35.28). The exclusion criteria were as follows: patients aged <20 years, patients who underwent both bioprosthetic and mechanical valve replacement, and patients who presented with lethal ventricular arrhythmias or who underwent an ICD implantation before the procedures. Patients underwent an ICD implantation according to the reimbursement criteria of National Health Insurance (NHI), including prior sudden cardiac arrest due to VTa, documented sustained VT/VF, or syncope caused by VA. ICD implantation was identified by the material codes of the NHI.

This study was approved in accordance with the Good Clinical Practice Guidelines by the Institutional Review Board of Taipei Veterans General Hospital.

### Databases and Characteristics of the Participants

The Taiwan Collaboration Center of Health Information Application, Ministry of Health and Welfare, provided all datasets of the NHIRD. Taiwan's NHI program enrolled 26 million people (covering 99% of the country's population), including data on outpatient visits, hospital care, prescribed medications, and the National Death Registry. We obtained permission from the National Research Institute for the Department of Health and the Health Promotion Administration, Ministry of Health and Welfare.

The underlying diseases were identified according to the International Classification of Diseases, 9th Revision—Clinical Modification (ICD 9-CM) codes. To be coded for the study, the diagnosis must have been recorded twice in the outpatient records or at least once in the inpatient records. By linking to the NHIRD, we identified the following clinical variables: age, sex, type of valve replacement, number of valve replacements, history of diabetes mellitus, hypertension, chronic obstructive pulmonary disease, end-stage renal disease (ESRD) (5), congestive heart failure (CHF), coronary artery disease, and stroke.

### Outcomes During Long-Term Follow-Up

The outcomes assessed in this study were all-cause mortality, cardiovascular (CV) death (ICD 9-CM codes 390–429), VTa- (ICD 9-CM codes 427.1 and 427.4), CHF- (ICD 9-CM code 428), stroke- (ICD 9-CM codes 430–438), and atrial fibrillation (AF)-related hospitalization (ICD 9-CM code 427.31). VTa was defined as ventricular tachycardia (ICD 9-CM code 427.1), including both sustained (>30 s) or non-sustained VT (≥3 consecutive complexes), and ventricular fibrillation (ICD 9-CM code 427.4). Death was confirmed using data from Taiwan's National Death Registry. Follow-up was terminated in cases of death or if the patients lived beyond December 31, 2016.

### Statistical Analysis

Normally distributed continuous variables are presented as mean values and standard deviations. Student's *t*-test was used to compare differences between two groups. Categorical variables were expressed as numbers and percentages and were compared using the Chi-square test. The incidence rates of mortality, CV death, and hospitalization were calculated as the number of cases per 1,000 person-years during follow-up. To minimize the influence of confounding factors on the clinical outcomes, propensity scores (PS) were utilized to match patients with mechanical valve and bioprosthetic valve replacements. A one-to-one matching of pairs was conducted using identical propensity scores with a 0.15-caliper width for age, sex, hypertension, diabetes mellitus, chronic obstructive pulmonary disease, CHF, stroke, ESRD, and total site(s) of valve replacement.

The matched (conditional) Cox proportional-hazards regression model was utilized to compare the hazard ratios (HRs) with 95% confidence intervals (CIs) of the outcomes. Potential confounders were adjusted using two models (Model 1, adjusted for age and sex; Model 2, adjusted for Model 1 factors plus total site(s) of valve replacement, hypertension, diabetes mellitus, CHF, coronary artery diseases, chronic obstructive pulmonary disease, prior stroke, and ESRD). The level of statistical significance was set at a two-tailed alpha level of <0.05. The analyses were performed using SAS software (version 9.4, SAS Institute, Cary, NC, USA).

## Results

### Selection and Characteristics of the Study Population

**Figure 1:**
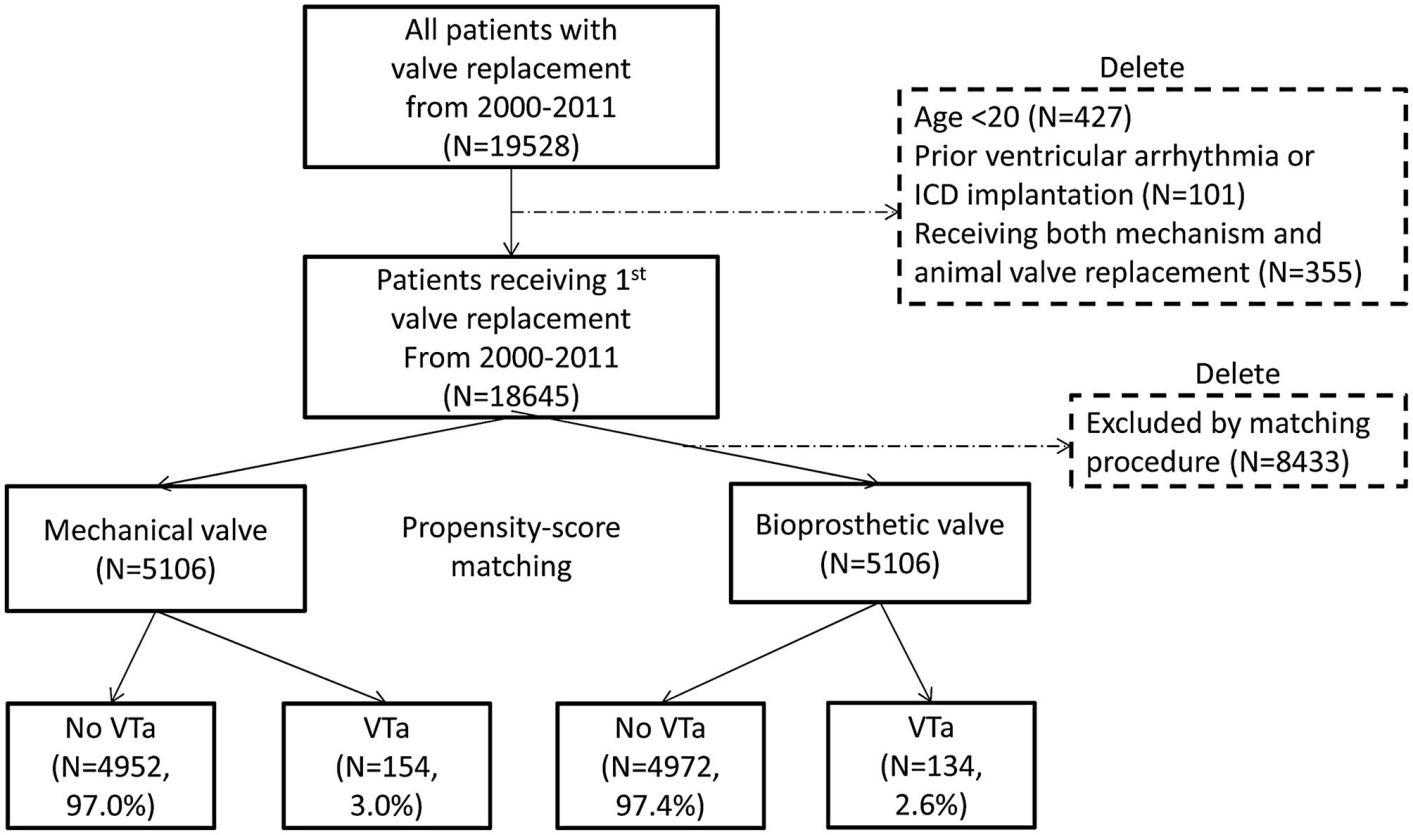
Flow chart of the present study. The selection process of study population and propensity score matching is presented. ICD, implantable cardioverter-defibrillator; VTa, ventricular tachyarrhythmia.

In total, 19,528 patients who had undergone their first valve replacement were identified in the NHIRD. After excluding 883 patients, 18,645 were included in the original cohort, and 10,212 patients were selected after propensity score matching (PSM). After PSM, both the mechanical valve and bioprosthetic valve groups had an equal number of 5,106 patients (Figure 1).

Table 1 shows the baseline characteristics of the original and PSM cohorts. After PSM, more patients underwent bioprosthetic valve replacement for aortic, tricuspid, and pulmonary valves (*P* = 0.04, *P* < 0.001, and *P* < 0.001, respectively), while more patients underwent mechanical valve replacement for mitral valves (*P* < 0.001).

**Table 1:** Baseline characteristics of the original cohort and propensity-score matched cohort.

**Variables**	**Original cohort (Total** ***N*** **=** **18,645)**			**Propensity-score matched cohort (Total** ***N*** **=** **10,212)**		
	**Mechanical valve (*N* = 13,537)**	**Bioprosthetic valve (*N* = 5,108)**	***P*-value**	**Mechanical valve (*N* = 5,106)**	**Bioprosthetic valve (*N* = 5,106)**	***P*-value**
Age	58.4 ± 14.6	64.7 ± 13.4	<0.001	64.7 ± 13.5	64.7 ± 13.4	0.98
Male sex	7,947 (58.7%)	2,883 (56.4%)	0.005	2,857 (56.0%)	2,883 (56.5%)	0.60
**Valve location**						
Aortic valve	7,497 (55.4%)	2,966 (58.1%)	0.001	2,859 (56.0%)	2,964 (58.0%)	0.04
Mitral valve	7,460 (55.1%)	2,500 (48.9%)	<0.001	2,697 (52.8%)	2,500 (49.0%)	<0.001
Tricuspid valve	497 (3.7%)	239 (4.7%)	0.002	159 (3.1%)	239 (4.7%)	<0.001
Pulmonary valve	38 (0.3%)	49 (1.0%)	<0.001	13 (0.3%)	49 (1.0%)	<0.001
Total site(s) of valve replacement	1.14 ± 0.36	1.13 ± 0.35	0.002	1.12 ± 0.34	1.13 ± 0.35	0.49
ICD implantation	66 (0.5%)	22 (0.4%)	0.61	21 (0.4%)	22 (0.4%)	0.88
**Underlying diseases**						
Diabetes mellitus (%)	597 (4.3%)	223 (4.4%)	0.79	200 (3.9%)	223 (4.4%)	0.25
Hypertension (%)	1,582 (11.7%)	596 (11.7%)	0.97	572 (11.2%)	596 (11.7%)	0.46
COPD (%)	218 (1.6%)	110 (2.2%)	0.01	95 (1.9%)	110 (2.2%)	0.29
Congestive heart failure (%)	3,028 (22.4%)	1,345 (26.3%)	<0.001	1,350 (26.4%)	1,343 (26.3%)	0.88
Prior stroke (%)	468 (3.6%)	199 (3.9%)	0.35	183 (3.6%)	199 (3.9%)	0.40
Coronary artery disease (%)	472 (3.5%)	184 (3.6%)	0.70	166 (3.3%)	184 (3.6%)	0.33
End-stage renal disease (%)	1,091 (8.1%)	461 (9.0%)	0.03	470 (9.2%)	461 (9.0%)	0.76

*COPD, chronic obstructive pulmonary disease; ICD, implantable cardioverter-defibrillator*.

### Ventricular Tachyarrhythmias After Surgical Valve Replacement

**Figure 2:**
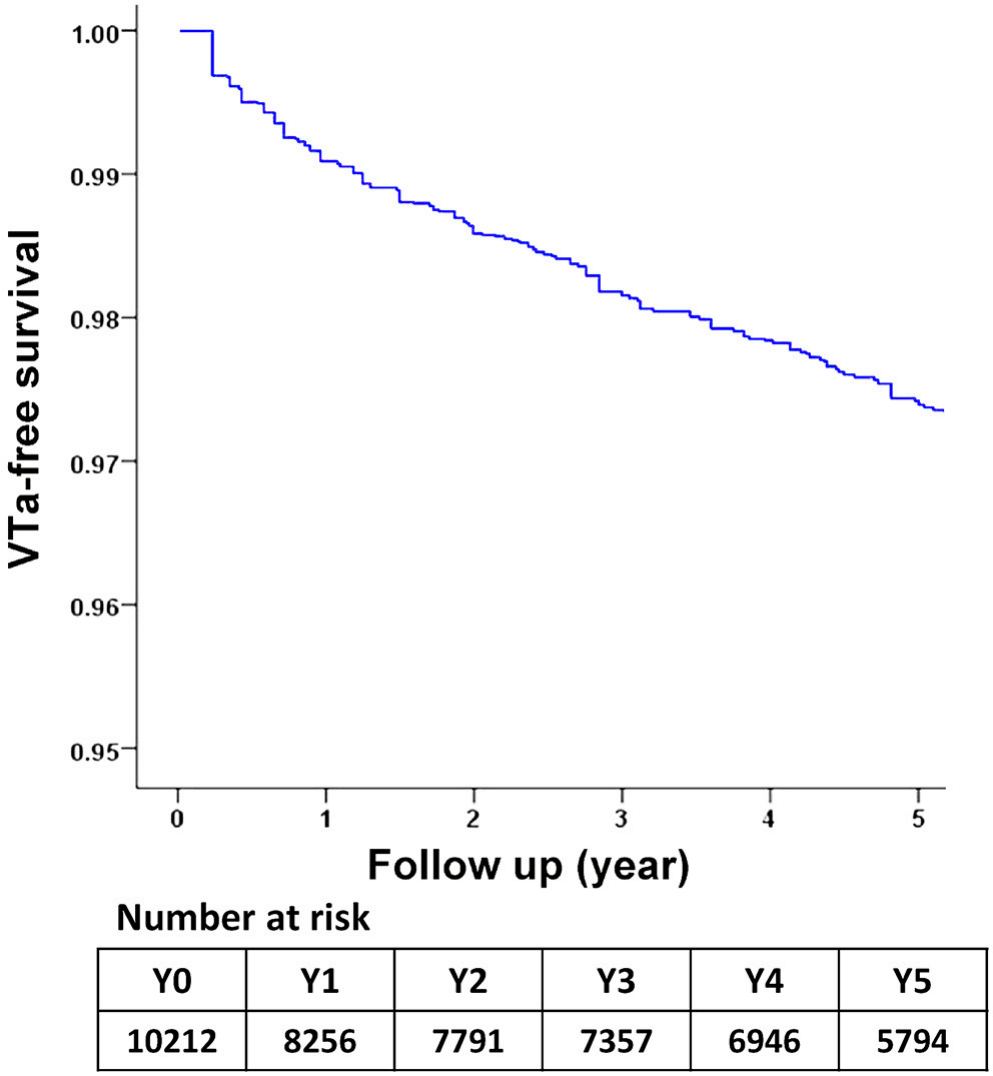
The survival curve of freedom from VTa after surgical valve replacement during 5-year follow-up. VTa, ventricular tachyarrhythmia.

During a median follow-up period of 59.6 months (25–75%, interquartile range 22.8–108.9), 288 (2.8%) patients with newly diagnosed VTa were identified, including 154 (1.5%) and 134 (1.3%) patients receiving surgical mechanical and bioprosthetic valve replacement, respectively (*P* = 0.23, Figure 1). The overall crude incidence rate of newly diagnosed VTa was 4.8/1,000 person-years. Among them, 4.8, and 4.7/1,000 person-years were identified in patients undergoing mechanical and bioprosthetic valve replacement, respectively (Table 2). Figure 2 shows the VTa-free survival curve after surgical valve replacement. After the early occurrence of VTa, the cumulative incidence of VTa persistently increased in the years following surgery.

**Table 2:** Incidence rates of subsequent events in various groups.

**Events**	**Groups**	**Total number**	**Total event number (%)**	**Person-years (PY)**	**Incidence rate (per 1000 PYs)**
VTa-related hospitalization	Mechanical valve	5,106	154 (3.0%)	32,229	4.8
	Bioprosthetic valve	5,106	134 (2.6%)	28,299	4.7
	Total	10,212	288 (2.8%)	60,528	4.8
All-cause mortality	Without VTa	9,924	5,323 (53.6%)	59,194	89.9
	With VTa	288	224 (77.8%)	1,740	128.8
	Total	10,212	5,547 (54.3%)	60,934	91.0
CV death	Without VTa	9,924	2,765 (27.9%)	59,194	46.7
	With VTa	288	140 (48.6%)	1,740	80.5
	Total	10,212	2,905 (28.4%)	60,934	47.7

*CV, cardiovascular; VTa, ventricular tachyarrhythmia*.

After adjusting for the effects of age, sex, total site(s) of valve replacement, and underlying disease using a multivariate regression analysis, bioprosthetic valve replacement was significantly associated with a lower risk of hospitalization related to VTa (*P* = 0.009, HR 0.63, 95% CI 0.45–0.89) than those who underwent mechanical valve replacement during the 5-year follow-up (Table 3).

**Table 3:** Comparison of risk of ventricular tachyarrhythmias between bioprosthetic and mechanical valve replacement.

**Groups**	**Risk factors**	**Mechanical valve (ref) vs. bioprosthetic valve**	
		**Hazard ratio (95% CI)**	***P*-value**
VTa-related hospitalization	Model 0	0.62 (0.44–0.88)	0.007
	Model 1	0.63 (0.44–0.89)	0.008
	Model 2	0.63 (0.45–0.89)	0.009

*Model 0: crude effect*.

*Model 1: Model 0 plus age and sex*.

*Model 2: Model 1 plus total site(s) of valve replacement, hypertension, diabetes mellitus, congestive heart failure, coronary artery diseases, chronic obstructive pulmonary disease, prior stroke, and end-stage renal disease*.

*VTa, ventricular tachyarrhythmia*.

Patients with VTa after surgical valve replacement more likely undergo mitral and tricuspid valve replacement (59.0 vs. 50.7%, *P* = 0.01; 7.3 vs. 3.8%, *P* = 0.003, respectively), less likely undergo aortic valve replacement (49.3% vs. 57.2%, *P* = 0.01), more likely undergo ICD implantation (11.1 vs. 0.1%, *P* < 0.001), and have a history of CHF (32.6 vs. 26.2%, *P* = 0.01) (Table 4).

**Table 4:** Baseline characteristics of patients with and without VTa after valve replacement.

**Variables**	**After propensity-score matching (Total** ***N*** **=** **10,212)**		
	**Without VTa (*N* = 9,924)**	**With VTa (*N* = 288)**	***P*-value**
Age	64.7 ± 13.5	65.4 ± 12.0	0.33
Male sex	5,588 (56.3%)	152 (52.8%)	0.23
**Valve location**			
Aortic valve	5,681 (57.2%)	142 (49.3%)	0.01
Mitral valve	5,027 (50.7%)	170 (59.0%)	0.01
Tricuspid valve	377 (3.8%)	21 (7.3%)	0.003
Pulmonary valve	59 (0.6%)	3 (1.0%)	0.34
Total site(s) of valve replacement	1.12 ± 0.34	1.17 ± 0.38	0.06
ICD implantation	11 (0.1%)	32 (11.1%)	<0.001
**Underlying diseases**			
Diabetes mellitus (%)	410 (4.1%)	13 (4.5%)	0.75
Hypertension (%)	1,142 (11.5%)	26 (9.0%)	0.19
COPD (%)	199 (2.0%)	6 (2.1%)	0.93
Congestive heart failure (%)	2,599 (26.2%)	94 (32.6%)	0.01
Prior stroke (%)	372 (3.7%)	10 (3.5%)	0.81
Coronary artery disease (%)	342 (3.4%)	8 (2.8%)	0.54
End-stage renal disease (%)	912 (9.2%)	19 (6.6%)	0.13

*COPD, chronic obstructive pulmonary disease; ICD, implantable cardioverter-defibrillator; VTa, ventricular tachyarrhythmia*.

### Effect of Ventricular Tachyarrhythmias on Mortality and Hospitalization

During long-term follow-up, the overall crude incidence rates of all-cause mortality and CV death after valve replacement were 91.0 and 47.7/1,000 person-years, respectively. Moreover, the crude incidence rates of all-cause mortality after valve replacement in patients with and without VTa were 128.8 and 89.9/1,000 person-years, respectively, while the crude incidence rates of CV death after valve replacement in patients with and without VTa were 80.5 and 46.7/1,000 person-years, respectively (Table 2).

Compared with patients without VTa after surgical valve replacement using a multivariate regression analysis, patients with VTa were associated with a higher risk of CV death (*P* < 0.001, HR 1.67, 95% CI 1.41–1.96), stroke- (*P* < 0.001, HR 1.66, 95% CI 1.37–2.01), AF- (*P* < 0.001, HR 2.80, 95% CI 2.42–3.24), and CHF-related hospitalization (*P* < 0.001, HR 2.61, 95% CI 2.30–2.95) (Table 5). Moreover, a similar risk of all-cause mortality, CV death, stroke-, AF-, and CHF-related hospitalization were observed between patients with VTa after surgical mechanical and bioprosthetic valve replacement (Supplementary Table 1).

**Table 5:** Comparison of various outcomes between patients with and without VTa after valve replacement.

**Groups**	**Risk factors**	**Without VTa (ref) vs. with VTa**	
		**Hazard ratio (95% CI)**	***P*-value**
All-cause mortality	Model 0	1.43 (1.28–1.58)	<0.001
	Model 1	1.39 (1.22–1.59)	<0.001
	Model 2	1.01 (0.89–1.15)	0.84
CV death	Model 0	1.23 (1.04–1.45)	0.02
	Model 1	1.66 (1.42–1.94)	<0.001
	Model 2	1.67 (1.41–1.96)	<0.001
Stroke-related hospitalization	Model 0	1.70 (1.40–2.06)	<0.001
	Model 1	1.67 (1.37–2.02)	<0.001
	Model 2	1.66 (1.37–2.01)	<0.001
AF-related hospitalization	Model 0	2.89 (2.51–3.34)	<0.001
	Model 1	2.83 (2.45–3.28)	<0.001
	Model 2	2.80 (2.42–3.24)	<0.001
CHF-related hospitalization	Model 0	2.71 (2.40–3.06)	<0.001
	Model 1	2.61 (2.29–3.00)	<0.001
	Model 2	2.61 (2.30–2.95)	<0.001

*Model 0: crude effect*.

*Model 1: Model 0 plus age and sex*.

*Model 2: Model 1 plus total site(s) of valve replacement, hypertension, diabetes mellitus, congestive heart failure, coronary artery diseases, chronic obstructive pulmonary disease, prior stroke, and end-stage renal disease*.

*AF, atrial fibrillation; CHF, congestive heart failure; CV, cardiovascular; VTa, ventricular tachyarrhythmia*.

In addition, lower all-cause mortality and CV death after VTa were observed in patients with ICD implantation after surgical valve replacement than in those without ICD implantation (*P* < 0.001, HR 0.35, 95% CI 0.20–0.59; *P* = 0.002, HR 0.34, 95% CI 0.17–0.66, respectively; Table 6). However, the incidences of stroke-, AF-, and CHF-related hospitalization were comparable in patients experiencing VTa with and without ICD implantation after surgical valve replacement (Table 6).

**Table 6:** Comparison of various outcomes between patients experiencing VTa with and without ICD implantation.

**Groups**	**Risk factors**	**Without ICD (ref) vs. with ICD**	
		**Hazard ratio (95% CI)**	***P*-value**
All-cause mortality	Model 0	0.32 (0.19–0.56)	<0.001
	Model 1	0.34 (0.20–0.57)	<0.001
	Model 2	0.35 (0.20–0.59)	<0.001
CV death	Model 0	0.32 (0.16–0.64)	0.001
	Model 1	0.33 (0.17–0.63)	0.001
	Model 2	0.34 (0.17–0.66)	0.002
Stroke-related hospitalization	Model 0	0.64 (0.32–1.25)	0.19
	Model 1	0.65 (0.33–1.26)	0.20
	Model 2	0.71 (0.36–1.40)	0.32
AF-related hospitalization	Model 0	0.96 (0.58-1.59)	0.86
	Model 1	1.09 (0.65–1.81)	0.74
	Model 2	0.99 (0.59–1.66)	0.98
CHF-related hospitalization	Model 0	1.04 (0.67–1.59)	0.88
	Model 1	1.07 (0.70–1.64)	0.74
	Model 2	1.24 (0.83–1.88)	0.30

*Model 0: crude effect*.

*Model 1: Model 0 plus age and sex*.

*Model 2: Model 1 plus total site(s) of valve replacement, hypertension, diabetes mellitus, congestive heart failure, coronary artery diseases, chronic obstructive pulmonary disease, prior stroke, and end-stage renal disease*.

*AF, atrial fibrillation; CHF, congestive heart failure; CV, cardiovascular; ICD, implantable cardioverter-defibrillator; VTa, ventricular tachyarrhythmia*.

## Discussion

### Major Findings

This study has several notable findings. First, the crude incidence rate of newly diagnosed VTa after surgical valve replacement was 4.8/1,000 person-years during 5-year follow-up. In addition to the early occurrence of VTa after surgery, the cumulative incidence of VTa persistently increased in the following years. Second, compared with patients without VTa, patients with VTa after surgical valve replacement were associated with a higher risk of CV death, stroke-, AF-, and CHF-related hospitalization. Third, lower all-cause mortality and CV death after VTa were observed in patients undergoing ICD implantation after surgical valve replacement, reflecting the importance of evaluating the need for ICD implantation in these patients.

### Ventricular Tachyarrhythmias in Patients Undergoing Surgical Valve Replacement

VTa frequently contributes to poor outcomes in patients with VHD, including reduced quality of life and increased risk of sudden cardiac arrest (1). Despite surgical replacement of VHD, several studies have reported that patients with VHD are still at risk for VTa even after valve replacement (4, 6–8). Konishi et al. reported that multiple valve replacement and larger heart size were associated with late occurrences of ventricular arrhythmias among long-term survivors who underwent aortic and/or mitral valve replacement (9). Similarly, in the present study, there was a trend toward more sites of surgical valve replacement in patients with VTa than those without (Table 4). On the other hand, patients with VTa after surgical valve replacement more likely undergo mitral or tricuspid valve replacement and less likely undergo aortic valve replacement, implying that different sites of valvular replacement may contribute to ventricular arrhythmogenesis to a varying degree. However, the above findings and the associated mechanism require further validation and investigation.

Possible mechanisms of VTa in patients with VHD could be multifactorial. VT caused by bundle branch reentry (BBR) has been reported in patients with VHD, both before and after valve replacement (10–14). Narasimhan et al. demonstrated that BBR accounts for 30% of sustained monomorphic VT cases after valve surgery (15). It might be due to an impairment of the conduction system during surgery and, therefore, emerges as the most common type of VT in the early postoperative period. In addition, the postulated precipitating factors for ventricular arrhythmogenesis include invading calcification from the degenerated valve to the conduction system, mechanical irritation to the myocardium by the prosthetic valve, ischemia, heightened adrenergic state of the postoperative period, and surgical scar (2, 13, 16–20).

The underlying diseased ventricular substrates in VHD may also increase the risk of VTa, which could be supported by the present finding of a higher incidence of CHF in those with VTa after surgical valve replacement. The correlation between VTa and left ventricular (LV) dysfunction in patients with VHD has also been reported in several studies (21–27). Chronic pressure or volume overload of the myocardium could increase ventricular wall stress and structural remodeling, which subsequently enhances the formation of arrhythmogenic substrates even after surgical valve replacement, despite the fact that regression of ventricular arrhythmia in consonance with the improvement of LV systolic function has been reported after valve replacement (28–30).

Michel et al. reported an increased frequency and worsening of ventricular arrhythmias early after aortic valve replacement (31). The early occurrence of VTa after valve replacement was also higher in the present study (Figure 2). However, the cumulative incidence of VTa persistently increased in subsequent years, which is consistent with the abovementioned mechanism of VTa. In addition to acute operative injury and mechanical irritation, chronic structural remodeling and diseased substrates also play roles in arrhythmogenesis and continue to increase the risk of VTa in the late postoperative stage.

### Influence of Ventricular Tachyarrhythmias and ICD Implantation After Surgical Valve Replacement

Olafiranye et al. reported that non-sustained VT documented by 24-h ambulatory electrocardiography within 18 months after mitral valve surgery was associated with late postoperative cardiac mortality (32). Notably, sudden cardiac death is considered an important cause of cardiac mortality in these patients. Moreover, Hochreiter et al. demonstrated that most postoperative sudden deaths were observed in patients with documented non-sustained VT by routine Holter monitoring after surgery for chronic non-ischemic mitral regurgitation (33). Consistent with the above findings, we found that the presence of VTa was associated with various worse outcomes. Irrespective of the underlying VHD, coexistent coronary artery disease, intraoperative injuries, and LV dysfunction may all play a role in the increased mortality and morbidity rate in patients with VTa after surgical valve replacement (15, 33).

Moreover, lower all-cause mortality and CV death after VTa were observed in patients with ICD implantation after surgical valve replacement than in those without, reflecting the potential role of ICD in the prevention of fatal ventricular arrhythmia in these high-risk patients. However, future studies are warranted to investigate potential surrogate markers for risk stratification of future fatal arrhythmias in patients who have not fulfilled the current recommendations for ICD implantation.

### Strengths and Limitations

To the best of our knowledge, this is the first large-scale study to investigate the clinical impact of VTa on various outcomes after surgical valve replacement using a nationwide population-based database. In addition, this study highlighted the importance of risk assessment for ICD implantation in patients with documented VTa after surgical valve replacement.

Nevertheless, this study has some limitations. First, given the retrospective design, some inevitable biases exist in this study. Second, despite PSM, some variables between the mechanical and bioprosthetic valve groups remained inconsistent. Third, diagnostic and procedural coding errors might exist. Fourth, some important parameters, such as left ventricular ejection fraction during peri-procedural period, extent of CAD, or detailed characteristics of VTa, cannot be extracted from the National Health Insurance Research Database. In addition, some detailed information was not available in the present study, including the pre-procedural between-group distribution of AF, or concomitant CABG in valve replacement. Future prospective researches will be warranted to clarify the above concerns. Finally, this study does not explore surrogate markers for future risk stratification and identify candidates for ICD implantation in addition to current recommendations. Future investigations are needed to elucidate the high-risk population in patients with VTa after surgical valve replacement.

## Conclusions

The crude incidence rate of VTa after surgical valve replacement was 4.8/1,000 person-years, which is attributed to both early and late postoperative VTa. The presence of VTa after surgical valve replacement significantly led to higher CV death and hospitalization rates due to various causes, while ICD implantation reduced the mortality rate in these patients.

## Data Availability Statement

The raw data supporting the conclusions of this article will be made available by the authors, without undue reservation.

## Author Contributions

All authors listed have made a substantial, direct and intellectual contribution to the work, and approved it for publication.

## Conflict of Interest

The authors declare that the research was conducted in the absence of any commercial or financial relationships that could be construed as a potential conflict of interest.

## Abbreviations

AF: atrial fibrillation
BBR: bundle branch reentry
CHF: congestive heart failure
CI: confidence interval
CV: cardiovascular
ESRD: end-stage renal disease
HR: hazard ratio
ICD: implantable cardioverter-defibrillator
LV: left ventricular
NHIRD: National Health Insurance Research Database
PSM: propensity-score matching
VF: ventricular fibrillation
VHD: valvular heart disease
VT: ventricular tachycardia
VTa: ventricular tachyarrhythmia.
