# Supplementary material for: Clinical Significance of Ventricular Tachyarrhythmias in Patients Undergoing Valve Replacement: A Nationwide Population-Based Study
Source: Front Cardiovasc Med. 2021 Jul 15;8:676897. doi: 10.3389/fcvm.2021.676897 (PMC8319543; doi:10.3389/fcvm.2021.676897)
Supplement: Supplementary file 1 [file Table_1.DOCX]

| **Supplementary Table 1. Comparison of various outcomes between bioprosthetic and mechanical valve replacement in patients experiencing VTa after valve replacement** | | | |
| --- | --- | --- | --- |
| **Groups** | **Risk factors** | **Mechanical valve (ref)**  **vs. bioprosthetic valve** | |
|  |  | **Hazard ratio (95% CI)** | **P-value** |
| **All-cause mortality** | **Model 0** | 1.01 (0.78-1.30) | 0.95 |
|  | **Model 1** | 0.95 (0.73-1.24) | 0.70 |
|  | **Model 2** | 0.99 (0.76-1.29) | 0.93 |
| **CV death** | **Model 0** | 0.92 (0.66-1.29) | 0.64 |
|  | **Model 1** | 0.89 (0.63-1.25) | 0.49 |
|  | **Model 2** | 0.94 (0.66-1.32) | 0.71 |
| **Stroke-related hospitalization** | **Model 0** | 0.96 (0.66-1.40) | 0.83 |
|  | **Model 1** | 0.97 (0.67-1.40) | 0.85 |
|  | **Model 2** | 1.07 (0.73-1.56) | 0.74 |
| **AF-related hospitalization** | **Model 0** | 0.83 (0.62-1.12) | 0.22 |
|  | **Model 1** | 0.82 (0.61-1.11) | 0.20 |
|  | **Model 2** | 0.80 (0.60-1.09) | 0.15 |
| **CHF-related hospitalization** | **Model 0** | 1.02 (0.79-1.31) | 0.88 |
|  | **Model 1** | 1.00 (0.78-1.29) | 0.98 |
|  | **Model 2** | 1.00 (0.78-1.29) | >0.99 |
| Model 0: crude effect  Model 1: Model 0 plus age and sex  Model 2: Model 1 plus total site(s) of valve replacement, hypertension, diabetes mellitus, congestive heart failure, coronary artery diseases, chronic obstructive pulmonary disease, prior stroke, and end-stage renal disease  AF = atrial fibrillation; CHF = congestive heart failure; CV = cardiovascular; VTa = ventricular tachyarrhythmia | | | |
